# Supplementary material for: Analysis of plasma proteins using 2D gels and novel fluorescent probes: in search of blood based biomarkers for Alzheimer’s disease
Source: Proteome Sci. 2022 Jan 26;20:2. doi: 10.1186/s12953-021-00185-9 (PMC8790928; doi:10.1186/s12953-021-00185-9)

**Supplemental Material.**

**Immuno-depletion and sub-fractionation of abundant plasma proteins**

*Plasma Pooling.* The plasma from 12 individuals, selected to be pooled together based on sex and disease status, were thawed on ice for 1 hour. The plasma was centrifuged at 16,500 relative centrifugal force (rcf) for 2 minutes and 50 µL from each individual was mixed together to generate the final pooled samples.

*Immuno-depletion.* For immuno-depletion, 45 µL of the pooled plasma samples was mixed with 135 µL of degassed Agilent buffer A. This was briefly vortexed and centrifuged at 2000 rcf for two minutes at room temperature. 160 µL of the supernatant was immediately loaded into the FPLC system for immuno-depletion (P-920 pump and UPC-900 UV detection at 280 nm, Amersham Biosciences, 2.0 mL PEEK tubing samples loop). Immuno-depletion was carried out with the multiple affinity removal system 14 column (MARS-14, 4.6x100mm, Agilent) following manufacturer’s instructions. The gradient used was precisely as described by the manufacturer. The MARS14 column targets serum albumin, transferrin, haptoglobin, IgG, IgA, α1-antitrypsin, fibrinogen, α2-macroglobulin, α1-acid glycoprotein, complement C3, IgM, apolipoprotein AI, apolipoprotein AII and transthyretin for depletion. The low abundance, flow-through proteins were collected in 15 mL conical vials between 2.4 and 9.4 minutes onto 0.82 g urea (SigmaUltra) and 23 µL glacial acetic acid (EMD Chemicals), for a final concentration of 6 M urea and 1% acetic acid. Solutions were gently mixed during the collection. After complete mixing, the flow-through solutions were frozen at --80° C until reverse-phase (RP) fractionation.

**RP-HPLC fractionation.** For HPLC sub-fractionation of the low abundance proteins, the collected MARS-14 flow-through protein/urea/acetic acid mixtures were thawed and loaded by two 1.15 ml injections using a manual analytical injector (Rheodyne), onto a C18 column (Agilent high-recovery macro-porous 4.6 mm X 50 mm). A binary pump, column heater and variable wavelength detector (Agilent) were used. The RP column temperature was maintained at 80° C. Mobile phase compositions were: A- MilliQ H_2_0 (Millipore)/ 0.1% TFA (Fisher Scientific), B- 95% acetonitrile (J.T. Baker HPLC grade)/ 0.08 % TFA at a flow-rate of 0.75 ml/min. A 30 s pause at 3.0 mins for the second manual injection was used. Table S1 details the gradient used for the fractionation of the low abundant proteins. Six sub-fractions were collected between minima from 9.0 min to 65.5 min (Figure S1) with the remainder of the method programmed for column cleaning. This was followed by a 20 min post-run of 3% B to re-equilibrate the column prior to the next injection. A large, uncollected peak that eluted at 66.5 min had low fluorescent character (excitation 280 nm, 330 nm emission) and is believed to be mostly non-proteinaceous lipemic material.

Fraction cut-points were chosen at minima in the HPLC UV trace (Figure S1) to yield approximately 200 μg of protein after the respective sub-fractions from six HPLC runs of each pool were collected, frozen and combined in a 50 mL conical vial (Falcon). After lyophilizing, the dried proteins were sequentially washed from the 50 ml vials, first with 40 μl of 8 M urea, 4% CHAPS (Sigma) followed by 1560 μL deionized H_2_0. The washes were combined in a 1.7 ml microfuge tube (Axygen), vortexed and divided into four 400 μL aliquots in 1.7 ml microfuge tubes. The aliquots were frozen and lyophilized to dryness, each yielding pellets of approximately 50 μg of protein suspended in a light matrix of urea and CHAPS. The pellets were stored at -80° C until fluorescent dye labelling.

**2D-PAGE**. 24 cm pH 3-11 non-linear Immobiline™ Drystrips (GE Healthcare) were passively rehydrated with 400 µL of rehydration buffer (8 M urea, 4% CHAPS, 1% Destreak™ (GE Healthcare), 1% pH 3-11 IPG buffer (GE Healthcare) and a trace bromophenol blue (Sigma-Aldrich), under biotechnology grade mineral oil (Bio-Rad), for nine hours at 4° C prior to isoelectric focusing.

For each of the six RP-HPLC sub-fractions, the samples were labelled with spectrally resolved fluorescent dyes (JAV-I-187, blue emitting; BDR-I227, green emitting) according to table S2. The use of spectrally resolved fluorescent dyes allows the independent analysis of two samples run on a single gel and greatly improves the digital superposition of the images during the spot matching step. To label the proteins, prepared pellets were dissolved in 7 µL of 45 mM bicine buffer (Calbiochem) pH 8.5, and briefly centrifuged at 1000 rcf. 1 µL of 1 mM dye in dimethylformamide (Thermo Scientific) was added and gently mixed. After brief centrifugation, the labelling reactions were placed on ice in the dark. After 30 minutes, unreacted dye was quenched with 1 µL of 50 mM lysine (Fluka) for 10 minutes. Samples that were combined on a gel according to table S2 were then mixed and 125 µL of rehydration buffer was added. The labelled protein mixtures were cathodic cup-loaded and isoelectric focusing was performed on an Ettan™ IPGphor II™ ceramic manifold according to the follow protocol: Step 500V for 1000 VHrs; Gradient 1000V for 5 hours; Gradient 8000V for 3 hours; Step 8000V for 3 hours.

After focusing, the strips were gently rocked in the dark for 15 minutes in 10 ml of 1% DTT (Bio-Rad) in equilibration buffer (6 M urea, 2% SDS, 20% glycerol, 50 mM Tris-HCl pH 8.8), followed by 15 minutes in 10 ml of 2.5% iodoacetamide (Sigma-Aldrich) in equilibration buffer.

For the second dimension PAGE, the focused strips were sealed in agarose with a trace of bromophenol blue onto 24cm X 1.5mm, 11% acrylamide gels. PAGE was run on a water-cooled DALT6 tank for 1 hour at 2 W per gel, followed by approximately 5 hours at 15 W per gel, until the blue dye front migrated to the bottom of the gels.

Gels were scanned for fluorescence using a Typhoon™ Trio scanner (GE Healthcare). Photomultiplier tube voltages were selected to assure sub-saturating intensities of protein spots except in the case of two abundant species in F3 and F5, that were later removed from the analyses. All scans were acquired at 100 µm resolution. Scans of the blue emitting JAV-I-187 labelled proteins used the 488 nm laser and an HQ 510 nm, 30 nm bandpass emission filter (Chroma Technology). For the green emitting BDR-I-227 labelled proteins, the 532 nm laser was used with a 580 nm, 30 nm bandpass emission filter (GE Healthcare). The chemical structures, excitation maxima (λ_max_) and emission maxima (Em_max_) are shown in Figure S2.

**Spot matching and quantitation.** Gel image files were imported into Progenesis SameSpots software (Nonlinear Dynamics V3.1.3030.23662). Gel image margins and saturating spots (intact VDBP in F3 and apolipoprotein AI in F5) were masked from the analyses. All images within a fraction were digitally aligned to match a representative “master” gel by entering up to 50 “seed” landmarks followed by automated spot matching and visual validation of the matches. After automatic spot detection and background removal, the images were edited manually using the spot adding, splitting and removing features of the software. Artefacts were removed from the analysis, and misalignments were corrected by manual warping by adding landmarks when appropriate.

**In-gel digestion.** Spots of interest were excised manually from analytical or preparative gels of 250-300 μg fractionated proteins. Preparative gels were visualized by preparing with Oriole fluorescent gel stain (Biorad) according to manufacturer instructions. Spots were picked on a UV transilluminator or Typhoon imager (GE Healthcare) and placed in 1.5 mL microfuge tubes (Eppendorf Protein LoBind). Gel-pieces were diced and washed/destained twice by vortexing for 10 minutes in 70 μl 50% acetonitrile (ACN, EMD Millipore-Omnisolv)/25 mM triethylammonium bicarbonate (TEAB, Sigma-Aldrich) and vacuum centrifuged until dry. 5-10 μL of 0.001% trypsin (w/v, Sigma-Aldrich proteomics grade porcine trypsin) in 50 mM TEAB was added to reconstitute the dried gel pieces and 50 mM TEAB was added to cover them. After digesting overnight at 37° C, supernatants were collected and peptides were extracted twice with 60 μL of 50% ACN/5% formic acid by vortexing and water-bath sonication for 5 minutes. The samples were concentrated by vacuum centrifugation to 10-15 μL.

**MS/MS and database search.** Trypsin digests were submitted for LC MS/MS analysis in positive ion mode by 1 of 3 methods (Table 3): Method #1- Run controlled by Agilent Chemstation software (Rev A.10.02), Nanochip module-LC ion-trap MS in CID mode. Agilent module and nanochip 43mm x 75μm Zorbax 300SB-C18 5μm RP separation column, Agilent 6330 XCT ion-trap, solvent A-100% H_2_O (J.T. Baker), 0.1% formic acid (FA, EMD Millipore-Suprapur), solvent B-100% ACN (EMD Millipore-OmniSolv), 0.1% FA, nano-pump flow rate-0.5 μL/min, LC method comprised 2% B at 0-2 min, gradient to 30% B at 18 min, gradient to 95% B at 20 min, 95% B at 20-25 min, gradient to 2% B at 27 min, 2% B at 27-32 min; Method #2- Run controlled by Agilent Masshunter Acquisition Workstation software B.04.00, Nanochip module-LC QTOF MS (Agilent module and nanochip 150mm x 75μm Zorbax 300SB-C18 5μm RP separation column, Agilent 6520 QTOF MS, same solvents as method #1, nano-pump flow rate-0.6 μL/min, LC method comprised 5% B at 0-2 min, gradient to 35% B at 18 min, gradient to 95% B at 20 min, 95% B at 20-25 min, gradient to 2% B at 27 min, 2% B at 27-32 min, product ions in MS were monitored in the range 300-1700 m/z and 25-1700 for MS/MS; Method #3-Eksigent ULTRA nanoflow LC fitted with a nanoflex chip cube to ABSCIEX 5600 qQTOF and cHiPLC Column ChromXP, C18-CL, 3μm, 120Å, separating column (Eksigent USA).  The ion source was an ABsciex nano ionspray III source (2400V) fitted with a 10µm emitter (New Objective). Peptides were eluted using a 5-50% acetonitrile/ 0.1% formic acid gradient over 25min.  Data dependent acquisition was performed on ions between the m/z range of 400 - 1600 with an intensity threshold of  >60cps and product ion scans that covered the m/z range of 100-1800.

MS data were used to search the Swissprot database using MASCOT version 2.4.1 (Matrix Science) (Perkins et al., 1999). A peptide tolerance of 50 ppm and fragment tolerance of 1 Da were used for MS method #1, and a peptide tolerance of 25 ppm and a fragment tolerance of 0.1 Da were used for MS methods #2 and #3. Variable carbamidomethylation of cysteine, oxidation of methionine, deamidation of asparagine and glutamine, and a single missed cleavage were allowed for the search, using 2+ and 3+ peptide charge states.

**Sample processing for quantitative LC-MS/MS analysis.** LiHeparin plasma was diluted 10x with doubly distilled water (20 µL plasma + 180 µL) and to a 20 µL aliquot of the 10x diluted plasma 42 µL of 9 M urea 20 mM dithiolthretol (DTT, Sigma) 300 mM Triethyl ammonium biocarbonate (TEAB) pH 8.0 was added the samples were vortexed and incubated at 60 ˚C for 60 minutes. After incubation 23 µL of 0.2 M iodoacetamide (Sigma) was added followed by incubation at 37 ˚C in the dark. The samples were then diluted with 333 µL of 100 mM TEAB before the addition of 25 µL of 1 mg mL^-1^ trypsin (Sigma). Digestion was allowed to occur overnight (16-18 hours) at 37˚C. Digestions were quenched by the addition of 5 µL of formic acid, followed by addition of the heavy stable isotope labeled peptide standards. Samples were desalted, using solid phase extraction C_18_ AssayMAP cartridges (Agilent Technologies), with the BravoAssayMap liquid handling system (Agilent Technologies).

Table S1.

| Time  (min) | %B |
| --- | --- |
| 0 | 3 |
| 4.5 | 3 |
| 5.5 | 30 |
| 52.5 | 48 |
| 62.5 | 100 |
| 66.5 | 100 |
| 67.5 | 3 |
| 68.5 | 3 |
| 70 | 60 |
| 77 | 100 |
| 79 | 100 |
| 80 | 3 |
| 100 | 3 |

Reverse phase HPLC gradient used for macroporous C18 separation of low abundant plasma proteins. Operated at 80˚C, 0.75mL * min^-1^.

Table S2. Gel fluorescent labeling and pooling schema used for each of the six RP fractions. MAD-male AD, FAD-female AD, MHC-male healthy control, FHC-female healthy control. Three independent pools (P) were prepared for each group.

| Zdye | GEL #1 | GEL #2 | GEL #3 | GEL #4 | GEL #5 | GEL #6 |
| --- | --- | --- | --- | --- | --- | --- |
| JAV-I-187 (blue emitting) | MAD P1 | FHC P2 | MAD P3 | MHC P1 | FAD P2 | MHC P3 |
| BDR-I-227 (green emitting) | FHC P1 | MAD P2 | FHC P3 | FAD P1 | MHC P2 | FAD P3 |

Table S3. Peptide precursor, collision energy and transition used to measure the abundance of peptides in figure 7.

**Figure legends:**

Figure S1. RP-HPLC UV chromatogram of MARS-14 immuno-depleted proteins with cut-points (dotted lines) at local minima used to collect six fractions for 2DGE. The two large uncollected peaks before F1 are the buffer flow-through and non-binding plasma components from two manual column loading injections. The large uncollected peak eluting after F6 has low intrinsic fluorescence (excitation 280/emission 330 nm) and is believed to be primarily lipids.

Figure S2. Structures of amine reactive blue emitting Zdye JAV-I-187 and green emitting Zdye BDR-I-227.

Figure S3. Example chromatograms of the peptide used to quantify the proteins in figure 7. The chromatogramss show the total intensity of the transitions monitored (Table S3) for either the endogenous light peptide (red), present in the plasma sample shown here, or the heavy labelled internal standard (blue).

Figure S1.


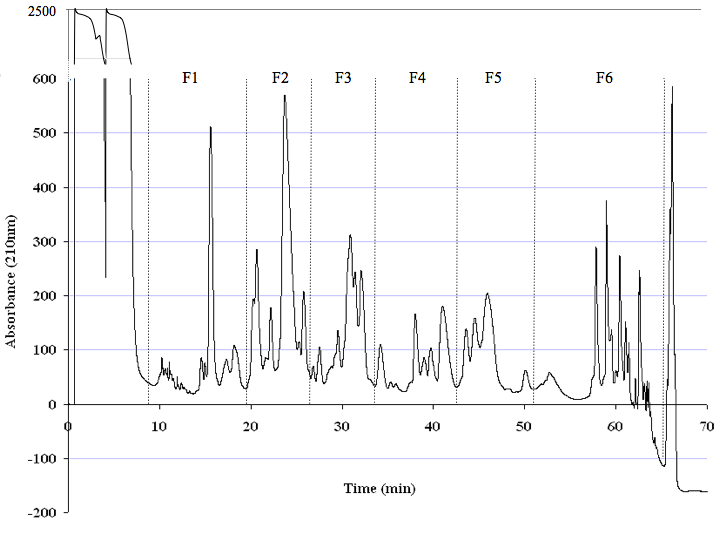


Figure S2.


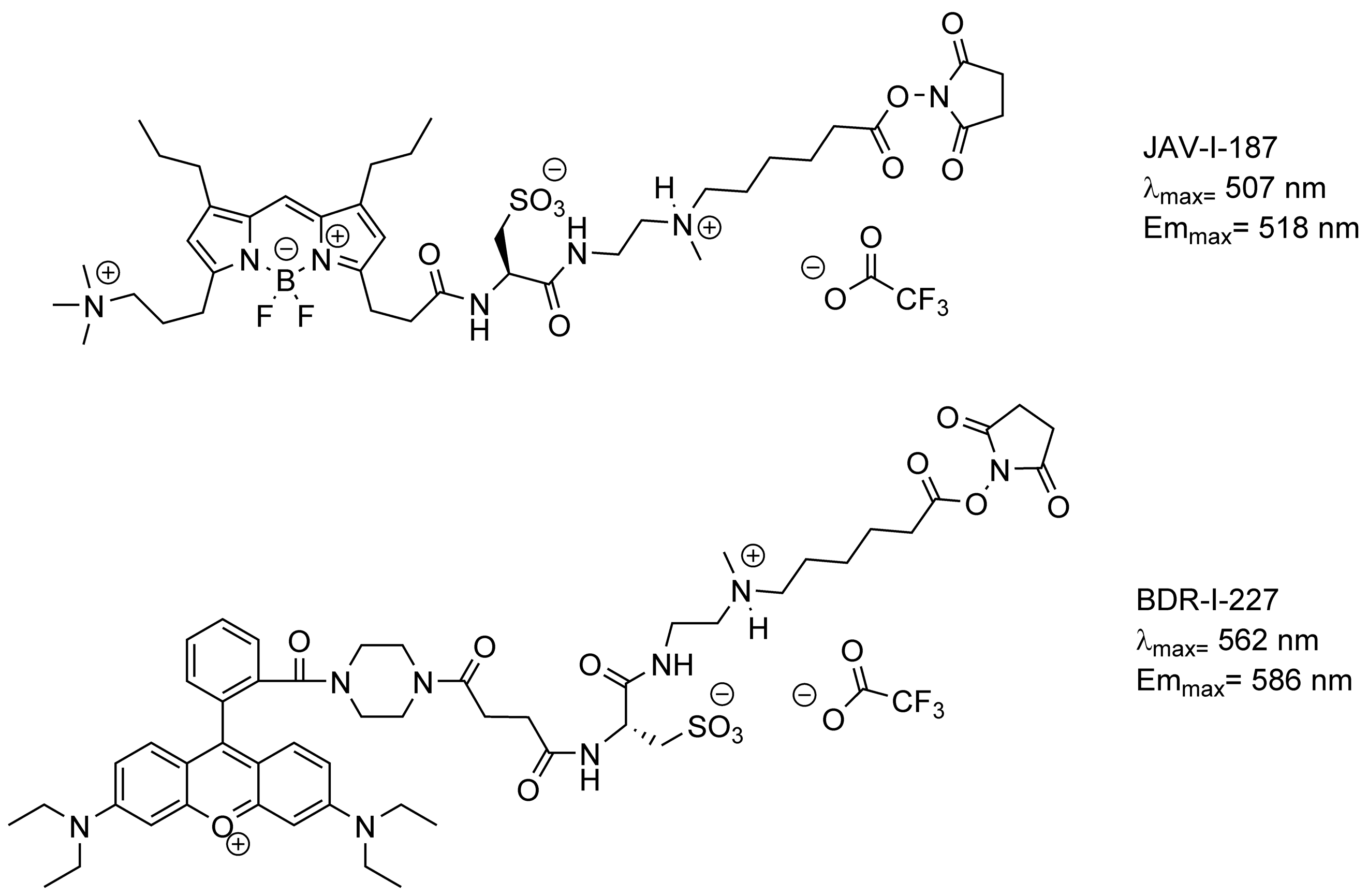


Figure S3.


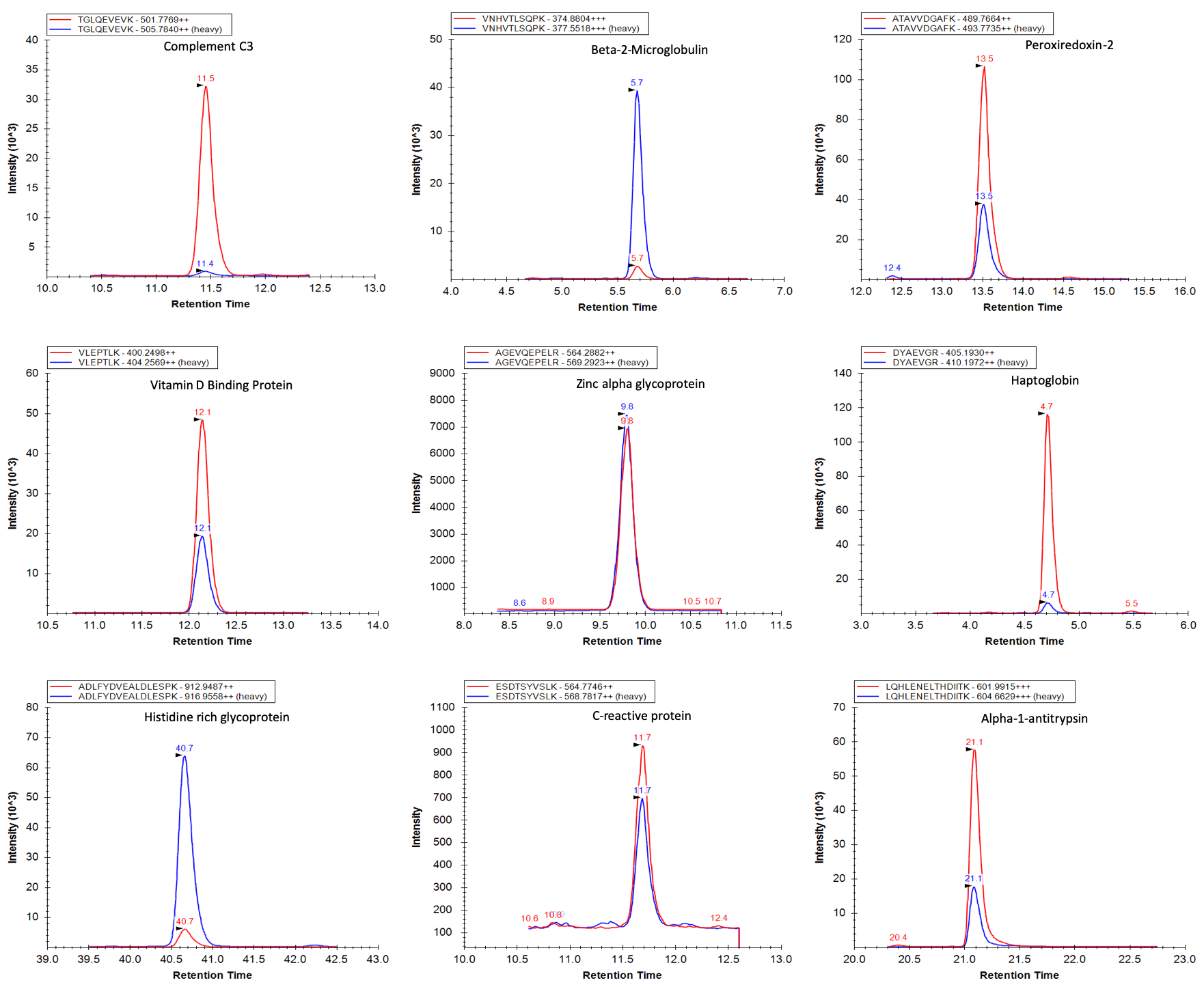

Supplement: Supplementary file 1 — Additional file 1. [file 12953_2021_185_MOESM1_ESM.docx]
